# What Works for You? Using Teacher Feedback to Inform Adaptations of Pivotal Response Training for Classroom Use

**DOI:** 10.1155/2012/709861

**Published:** 2012-11-18

**Authors:** Aubyn C. Stahmer, Jessica Suhrheinrich, Sarah Reed, Laura Schreibman

**Affiliations:** ^1^Child and Adolescent Services Research Center, Autism Discovery Institute, Rady Children's Hospital San Diego, 3020 Children's Way, San Diego, CA 92123, USA; ^2^Department of Psychology, University of California, San Diego, 9500 Gilman Drive, La Jolla, CA 92093-0109, USA

## Abstract

Several evidence-based practices (EBPs) have been identified as efficacious for the education of students with autism spectrum disorders (ASD). However, effectiveness research has rarely been conducted in schools and teachers express skepticism about the clinical utility of EBPs for the classroom. Innovative methods are needed to optimally adapt EBPs for community use. This study utilizes qualitative methods to identify perceived benefits and barriers of classroom implementation of a specific EBP for ASD, Pivotal Response Training (PRT). Teachers' perspectives on the components of PRT, use of PRT as a classroom intervention strategy, and barriers to the use of PRT were identified through guided discussion. Teachers found PRT valuable; however, they also found some components challenging. Specific teacher recommendations for adaptation and resource development are discussed. This process of obtaining qualitative feedback from frontline practitioners provides a generalizable model for researchers to collaborate with teachers to optimally promote EBPs for classroom use.

## 1. Introduction

One area of growing concern for both researchers and educators is providing educational services to children with autism spectrum disorders (ASD). Serving students with ASD poses a challenge to public schools because very few comprehensive interventions have been rigorously and systematically tested in school settings, as opposed to highly controlled research settings [1, 2]. Despite the limited data on the effectiveness of specific evidence-based practices (EBPs) in schools, many teachers report supporting and using such practices [3]. However, they also state that they typically modify EBPs for use in the classroom by combining and adapting EBPs from various training protocols to fit their own teaching preferences and the needs of individual students [4]. While it is possible that these informal adaptations based on teachers' judgment may not change the effectiveness of the intervention, research in other areas suggests that the positive outcomes demonstrated in research settings may not be maintained when programs are modified [5]. These issues are indicative of poor translation of information from research to educational settings.

Researchers and educators alike often are frustrated by the gap between research and practice [6–8]. There are concerns on both sides regarding the utility of EBPs developed in research settings for use in educational environments. Researchers question whether educators attempting to replicate research models adequately assess fidelity of implementation (the degree to which the intervention is used as specified) or replicate all aspects of the program [9]. Conversely, educators often feel that practices developed by researchers do not capture the richness and complexity of the children in their programs or the environments in which they work [3, 10]. It is imperative that this research-to-practice gap be closed through effective translation of EBPs into educational settings to improve the quality of available services for children with ASD.

Innovative models of intervention adaptation and implementation may provide a more effective response to the disconnect between research and practice. Such models shift from the traditional, unidirectional attempts to move information from research into practice settings toward a more reciprocal, interactive effort between researchers and practitioners [6, 11, 12]. To ensure that best practices are portable to usual care settings, researchers must gain a precise understanding of the perspectives of community stakeholders regarding the benefits and barriers of specific practices and work directly with stakeholders, such as teachers, to adapt interventions [13–15]. This type of collaboration will provide researchers a base from which to shape EBPs in a way that makes them feasible for use in community settings. Information gained from teachers can then be scientifically tested to ensure ongoing effectiveness of the intervention given recommended adaptations. Alternatively, if adaptations compromise effectiveness or alter child outcomes, new training methods or additional materials may be developed to better support teachers' ability to implement the original intervention with fidelity.

One EBP for students with ASD that can be used in classrooms is Pivotal Response Training (PRT). PRT is a naturalistic behavioral intervention, based on the principles of applied behavior analysis, which is soundly supported in the scientific literature [8, 16–18]. A recent review conducted by the National Professional Development Center on ASD listed PRT as one of 24 EBPs with evidence of efficacy for teaching students with ASD [19]. PRT is a multicomponent intervention shown to be efficacious for improving communication, play, academic skills, and social interaction. It is based on a series of studies identifying important treatment components and demonstrating their effect on child behavior. The “pivotal” responses trained in PRT are *motivation, initiation,* and *responsivity to multiple cues* (i.e., increasing breadth of attention). Specific elements include gaining attention, presenting clear and appropriate instructions, interspersing maintenance tasks, sharing control (including following the child's choice and taking turns), requiring the child to respond to multiple cues, providing contingent consequences, ensuring a direct relationship between the child's response, and the reinforcer and reinforcing attempts.

There is some evidence for the efficacy of PRT when implemented by classroom teachers [20–23] and paraprofessional staff [24]. However, these studies have been relatively small efficacy studies and therefore effectiveness of PRT is unknown when it is implemented by teachers who have not been trained in the context of a research protocol. Research examining usual care in the Southern California region indicates that more than seventy percent of 80 teachers surveyed reported using PRT or some variation of PRT in their classrooms. However, the majority reported adapting the intervention by using only certain parts or mixing PRT with other strategies (Stahmer, unpublished data; [25]). Some educational programs report using PRT in combination with other interventions [26–28], which may also affect treatment integrity and effectiveness. 

Several factors make PRT an excellent intervention for translation into school settings. Because PRT was developed for use in the natural environment, classrooms are an appropriate setting for PRT implementation, and there is evidence indicating teachers can learn to correctly implement PRT in one-to-one settings [29]. However, PRT was developed for use by clinicians and parents and has been tested primarily in individual sessions with one adult and one child. Special education teachers often teach in the context of small group activities and may not have the opportunity to work one-on-one with students during the majority of the school day. Therefore, there is a need to adapt PRT to better fit classroom settings. 

This study is part of a larger program designed to translate PRT into classrooms. The collaborative model of translation includes obtaining feedback from teachers, observing teachers' use of PRT in the classroom, testing recommended adaptations based on observation and feedback findings, and testing the modified program. The first step in the process of understanding how PRT may need to be adapted for effective use in the classroom is gathering information from teachers regarding their views on necessary adaptations. 

The purpose of this study was to work collaboratively with teachers to obtain their feedback on the benefits and barriers of using PRT in their programs as well as their recommendations for potential modifications. We used focus groups to gather teachers' input in order to obtain insight into how to increase the usability of PRT in applied settings. The process of gathering information from frontline stakeholders represents the first step in collaboration between teachers and researchers to effectively move EBPs for ASD into the classroom. 

## 2. Method

A focus group approach was chosen to obtain an understanding of the ways in which classroom teachers implement and modify existing PRT protocol. Focus groups are characterized by the use of participants who have a specific experience with or opinions about the topic and the exploration of subjective experiences of participants in relation to predetermined research questions [30, 31]. 

### 2.1. Participants

Focus groups were conducted with thirteen teachers serving children with ASD, aged three to eight, in San Diego County, who taught at least one student with ASD in their classrooms at the time of invitation. Invitations to participate in focus groups were made through phone calls and e-mails to 60 teachers serving the target population who had previously expressed general interest in participating in research studies. Interested teachers were asked to describe their knowledge and experience with PRT. Twenty of the teachers who were initially contacted were no longer working in the district or were no longer teaching special education at the time of contact. Of the 40 remaining teachers, 10 did not respond. Seven declined participation citing time constraints. Of the 23 teachers who expressed interest in participating, eight could not participate due to scheduling conflicts and two did not respond to scheduling phone calls. The remaining 13 were divided into three separate groups (based on self-report): (1) *PRT Trained and Using* (PRT-USE), teachers trained in PRT *and* currently using PRT (*n* = 5); (2) *PRT Trained and Not Using* (PRT-NO USE), teachers trained in PRT but *not *currently using PRT (*n* = 3); (3) *Not PRT Trained* (NOT TRAINED), teachers *not* trained in PRT (*n* = 5). All teachers who participated in the PRT-USE focus group reported either receiving on the job training (*n* = 3) or attending a PRT training workshop (*n* = 2). Training was provided by a supervisor (*n* = 3), classroom consultant (*n* = 1), or outside consultant (*n* = 1). In the PRT-NO USE group, one teacher reported attending a workshop, one reported receiving on the job training, and one reported attending a workshop and receiving on the job training from a district autism consultant. None of the teachers in the NO TRAINING group reported receiving PRT training of any kind. Consistent with the demographics of teachers serving students with ASD in San Diego, all participants were women. Of the 13 participating teachers, 12 (92%) were Caucasian, and 1 (8%) was Asian/Pacific Islander. Participants ranged in age from 28 to 65 years (*M* = 40.9, SD = 11.7). All 13 participants had a Bachelor's degree and 10 had Master's degrees. Teachers' years of experience working with children with ASD ranged from 1 to 34 years (*M* = 13.1, SD = 10.1). Teachers worked in 13 different schools, and classrooms included autism-only, cross-categorical, severe and mild/moderate designations.

### 2.2. Data Collection

An interview guide was developed to examine participants' perspectives on the use of PRT techniques in the classroom (see the Appendix for sample questions). Questions for the guide were generated based on the study goals and pilot discussion about their program methods with an advisory board of teachers and administrators serving children with ASD (see Section 2.4). Digital audio recordings were made using a Conference Grabber microphone and a laptop computer with Audacity recording software.

### 2.3. Procedure

Focus groups took place at a central location for all participants. Each group lasted no more than two hours. Informed consent was gathered at the start of the group, and participants completed a background questionnaire, which included demographics, teaching experience, and training/experience with PRT. 

 The moderator for all three groups followed the interview guide. After introductions, the moderator asked participants to provide an overview of their programs. Teachers in the NO TRAINING group were provided with a brief overview of PRT and each PRT component. Consistent with a well-established format in focus group methodology [32, 33], the next phase of the discussion used a vignette (see the Appendix for an excerpt of the vignette). A short vignette describing the hypothetical case history of a school age child with ASD was introduced to facilitate discussion among the teachers through exposure to uniform stimuli and to provide a basis for the quantification and comparison of responses within and across the focus groups. All participants received the same vignette. Participants were asked to read the vignette then decide what type of intervention they would recommend if such a student came to their program and how PRT might (or might not) be used. Subsequent questions focused on how participants would use PRT techniques or other strategies with the child described in the vignette, the setting and activities in which PRT might be used, and the general benefits and barriers to the use of these techniques in the classroom. To ensure that all participants provided input, the moderator asked different participants to begin each discussion and to provide input throughout the discussion. 

Next, the moderator asked participants to review a list of the specific components of PRT and discuss the benefits and barriers of each component, ease of use of each component, and whether they liked each component for classroom use. Teachers in the NOT TRAINED group were asked to discuss whether they used any similar strategies in their classrooms. At the end of each group, participants were thanked and given $20 as compensation for their participation. 

### 2.4. Data Analysis

Data analysis was guided by grounded theory (i.e., theory derived from data and then illustrated by characteristic examples of data; [34]). First, audio recordings of focus group discussions were transcribed by student research assistants, blind to the purpose of the project, and reviewed by the research team for accuracy. The transcripts were then independently coded by three of the project investigators at a general level in order to condense the data into analyzable units. The constant comparative method [34] was then used to identify five primary themes. Themes were identified by comparing codes assigned to segments of text and identifying characteristics shared among codes and characteristics that distinguished between codes. Segments of transcripts ranging from a phrase to several paragraphs were assigned codes based on a priori (i.e., based on questions in the interview guide) or emergent themes. Disagreements in assignment or description of codes were resolved through discussion and enhanced definition of codes. The final list of codes consisted of a list of themes, issues, accounts of behaviors, and responses to the presentations of the vignette. The transcripts were assessed for coding agreement between the authors using a procedure supported by other qualitative studies [35, 36]. To examine reliability, the number of coded statements on which the coders agreed was divided by the number of agreements plus disagreements and multiplied by 100. For all coded statements, the coders agreed 85% (range = 85-86%) of the time, indicating good reliability in qualitative research [35]. Disagreements were resolved through consensus. Disagreements typically arose when one coder missed an instance of a topic, typically when a teacher spoke about the principle of a PRT component generally (rather than naming the component itself). 

Themes were compared across groups to look for trends. A peer debriefing method [37] was used to provide some content validation. Results were presented to an advisory board of teachers and administrators familiar with PRT who provided feedback regarding interpretation of the teachers' comments. 

## 3. Results

Primary themes across the groups were identified as (a) the benefits of PRT; (b) barriers to use of PRT; (c) specific training issues; (d) recommendations for specific PRT components; (e) areas in need of empirical validation and adaptation. Themes were very similar across the three groups; therefore, data were collapsed. Representative quotes from various categories are provided as descriptive examples of the data. 

### 3.1. Benefits of PRT

 Teachers consistently reported that PRT made sense to them and fit well with their concepts of what constitutes good teaching for students with ASD.

*“You have items available that you know he's going to want so he's more likely to ask for it. He gets it and so he is reinforced for the desired behavior of requesting. It's good teaching.”*

They felt that PRT could be used successfully and appropriately with students who had a variety of disabilities, making it easy to implement in classrooms serving children with varied needs.

* “A lot of these are things all kids need, not just children with autism.”*

They found the components to be natural to use and consistent with good teaching practices. Teachers reported that PRT increased generalization of children's skills and found that parents viewed PRT positively.

*“I do have a hard time getting children to generalize, so I like having PRT because it helps that change over and it helps to bridge the gap.”*

### 3.2. Barriers to the Use of PRT

 Teachers reported some significant barriers to the use of PRT in their classrooms. In general, teachers found it easier to use more structured programs, such as Discrete Trial Teaching or TEACCH methods, due to ease of data collection and clarity of the procedures. Teachers reported that it was difficult to simultaneously take data and implement PRT correctly. While some teachers felt that PRT was very natural, others found that the requirement to follow a specific sequence of components was too controlling. In contrast, some teachers felt a lack of structure in PRT which made it difficult to keep the overall teaching sequence and components clear.

*“Because it really is loosely structured …I always feel like I am learning. I do not feel like an expert in any way. It gets a little confusing with all the pieces.”*

Teachers reported difficulty using PRT (or strategies similar to the PRT components) in group settings with multiple students.

*“Is there a way to develop this to use in a group? As opposed to having it be so one-on-one? Is there a way that you could use PRT with a group of three kids, four kids, five kids, so we could do it?”*

In addition, some teachers reported difficulty with implementation because PRT was not tied to a specific curriculum, which made it difficult to determine what goals to target or how to follow a child's individual education plan (IEP).

### 3.3. Specific Training Issues

Teachers in both trained groups reported that the lack of a clear and comprehensive PRT manual, or specific methods for using PRT in a classroom, made it difficult to train paraprofessionals (e.g., classroom assistants) in the intervention.

*“If you teach them [paraprofessionals] to do a certain piece, like interspersing maintenance tasks, they get the one, but if you try to do more than one it gets a little confusing and they are not sure what to focus on.”*

Teachers reported that certain prerequisite skills were needed to understand the foundation and goals of PRT. Specifically, they felt that paraprofessionals needed a basic understanding of the underlying principles of ABA in order to understand how and when to use PRT with students.

*“My hang up is really training. Last year when I did train I wanted to make sure they [paraprofessionals] were doing it correctly. I felt like it was harder for them to grasp unless they had that ABA background. So I needed to make sure they understood the basics of ABA before going on to PRT, otherwise it was just confusing.”*

Teachers stated that they needed a way to break down the components of PRT so they could teach paraprofessionals one or two components at a time rather than all at once. In addition, understanding how to explain the program in broadly accessible terms and having a simple way to communicate the method and supporting research to parents was important to them.

*“I've got a few parents I've talked to that love PRT, they think that it's just wonderful, and they want to do it at home. I wonder if it might be something we could offer to parents…if they understand how to use it and how it can help their children.”*

### 3.4. Recommendations Regarding Specific PRT Components

Teachers reported common perspectives on many of the individual components of PRT. The list of the components (with brief descriptions) and teachers' comments for each are summarized in Table 1. 

#### 3.4.1. Gaining Attention

There was clear consensus that gaining students' attention was important, but easy to forget.

*“If we are trying to promote language and play it [the instruction] should be clear, and the child must be attending in order to give an appropriate response.”*

Teachers expressed concerns that maintaining student attention (especially for students with ASD) in a group was not a realistic goal and that it was especially difficult for classroom assistants. 

#### 3.4.2. Presenting Clear Opportunities/Instructions

Teachers indicated that using clear instructions was consistent with their teaching strategies, and easy to implement consistently. They felt that using simpler language and clear instructions could be difficult for paraprofessionals.

*“I think using clear instructions is one of the most important [components] if you're going to get comprehension at all.”*

#### 3.4.3. Interspersing Maintenance Tasks

Teachers reported liking the use of maintenance tasks to keep student's frustration low but also indicated that this was a particularly difficult component to communicate to classroom staff.

*“One of the things I find frustrating is interspersing the maintenance tasks. I have to remind myself sometimes—you do not always have to expect this level—wait, remember to let them be successful this time….The two girls [paraprofessionals] who work in the classroom with me do not understand that. They are always looking for that one correct response…and then they wonder why we see some of the behaviors that we see. So I think that it is really important to remember that you've got to vary it [the task] and let them be successful and put those maintenance tasks in there.”*

#### 3.4.4. Using Shared Control (Following the Child's Choice and Taking Turns)

Teachers discussed the necessity of including child choice as way to improve students' motivation to participate. However, they felt that it was often unrealistic to allow children to consistently choose activities in the context of the classroom and particularly during group activities. Teachers found it difficult to take turns with the learning materials, particularly when the activity was not play based. Their typical teaching practices may involve a more didactic approach, or modeling an activity or lesson and then subsequently allowing the child to complete the activity.

* “There are times when it's appropriate for the child to make choices and take turns, and there are times when it is not, because that is the way the world is: There are times when you get to choose and times you do not.”*

#### 3.4.5. Requiring Response to Multiple Cues

Incorporating multiple cues or conditional discriminations (i.e., discriminations requiring response to two or more elements of a compound stimulus to make an appropriate response) is a component of PRT that has been shown to help broadening attention in children with autism [38–40]. For example, a student who is coloring may be offered a box of crayons and markers in different colors and instructed, “Choose a green crayon.” This instruction requires that the child choose something that is both green (not another color) and a crayon (not a marker) in order to make the appropriate response. Teachers reported that the use of multiple cues was particularly confusing and difficult to implement in the classroom due to the time it took to arrange the environment and materials to utilize these types of instructions.

* “For me it's too hard to have enough materials available in my classroom for each student and every goal to always be working on multiple cues.” *

In addition, teachers had concerns regarding the use of this component with students who they felt did not have the language or cognitive capacity to understand conditional discriminations. 

#### 3.4.6. Providing Contingent Consequences

Teachers agreed that contingent feedback is important and part of good teaching in general. However, they reported that it could be difficult if a child exhibits a correct behavior at a time or place that did not allow them to reward the behavior (e.g., a child with limited verbal skills spontaneously interrupts the teacher reading a story and requests bubbles).

*“Any response to a child's behavior must be contingent. But if it is circle time and he very appropriately and correctly requests to go outside, the answer is “no”, it's not time to go outside.”*

#### 3.4.7. Utilizing Direct Reinforcement

Teachers reported that direct reinforcement was an effective strategy in general. However, they indicated that the use of direct reinforcement was difficult for many skills taught in the classroom and for children who preferred edible reinforcers.

*“I basically let them choose their own reinforcer but there are times when I basically have to say, “No, you are not working for that reinforcer.” I know it is against everything we are supposed to be doing but I have kids that eat nothing but candy and I do not know what to do about it.”*

#### 3.4.8. Reinforcing Attempts

Teachers in all groups agreed that reinforcement of goal-directed attempts was important for maximizing student motivation. 

### 3.5. Areas in Need of Empirical Validation and Adaptation

Teachers were asked specifically about recommendations for adapting PRT for use in classroom environments. In particular, they reported a need for strategies that would allow them to use PRT in the context of their existing classroom structure. They also wanted to see specific techniques for implementing PRT in group settings of three or more students.

*“My classroom is not really set up to structure it for one particular child. It's not feasible with fifteen, twenty other kids in there.”*

Teachers reported using several other teaching strategies that either they felt were effective or that they were mandated to use by their district. Therefore, they felt it was important to know how to integrate PRT within these strategies.

*“There are five or six [interventions] I am supposed to be implementing in my program but it is understood that we should be taking pieces of each and incorporating them as we best see fit.”*

Teachers wanted information on how to individualize PRT for their students. Because special education classrooms focus on attainment of IEP goals for each student, they also requested information on how to use PRT to target students' IEP goals. Teachers reported that parents and schools are becoming “data driven” and asked for better data collection systems specific to PRT that would allow them to easily track and share student progress.

*“There is not a way to talk about child progress (with PRT). It's not very measurable, and so much of what we do is data driven.”*

In summary, teachers from focus groups representing three separate populations (PRT-USE; PRT-NOT USE and NOT TRAINED) had similar opinions on the specific components of PRT and how the intervention may be improved for use in the classroom. 

## 4. Discussion

 The results of this study provide preliminary data on teacher perspectives about how PRT may be adapted and supplemented to best fit classroom needs for students with ASD (see Table 2). There are several key findings that may lead to alterations to PRT that allow for more effective classroom implementation. Teachers reported valuing certain PRT components as important for student learning. Some of the valued components they found relatively easy to implement (e.g., presenting clear opportunities and reinforcing attempts), while others they found difficult (e.g., gaining attention, following the child's choice, utilizing contingent, and direct reinforcement), particularly in group settings. The components that teachers value and report no trouble implementing likely do not require adaptation, while more focused training may be needed to ensure appropriate implementation in the areas that are recognized as important but perceived as difficult. There are also some components teachers did not appear to value highly and also reported as difficult, such as taking turns and requiring a response to multiple cues. These components may require future research to test the effectiveness of adaptations, and/or teachers may need additional education regarding the necessity of these components for student learning. 

 These results are likely applicable to other naturalistic behavior methodologies using similar intervention components, including Incidental Teaching [41, 42], mand-model [43], time delay [44], and Milieu Teaching [45]. Although these specific techniques were developed in different laboratories, the approaches are similar in that they share many basic components. Examining teacher use of a variety of naturalistic strategies may lead to richer data regarding subtle differences in components and delivery strategies that may enhance or inhibit classroom implementation.

### 4.1. Additional Resources and Training

Additional or more intensive training in PRT in general may be helpful; however, training does not appear sufficient to overcome implementation barriers in this group of teachers. Some teachers who were trained in PRT were no longer using the intervention at all and a majority reported adapting the intervention. Therefore, adaptation and/or focused training based on teacher feedback are likely necessary. 

Providing contingent and direct consequences are two components of PRT that teachers find important but report as difficult to implement consistently. The next step toward effective translation of these components may be generating classroom-specific additional resources and strategies for implementation, so that teachers may more readily relate their training on these components to the day-to-day implementation of the strategy in their programs. Resources may include information on why providing contingent and direct consequences is important (based on supporting research) and how teachers can improve their use of contingent and direct consequences in the classroom context (with real-world examples, classroom scenarios, activity suggestions, and troubleshooting). In addition, creative methods of developing direct reinforcers that can be easily provided to multiple students in the classroom, for example, through token systems, may be explored for effectiveness. Similarly, teachers reported difficulty providing choices to students. Highlighting creative examples of providing choices within teacher-directed group learning contexts may decrease concerns regarding this component and perception of its difficulty. 

 Findings also indicated several areas of need for additional resources that are not related to the specific components of PRT. Teachers requested materials and strategies for training paraprofessionals, communicating with parents, collecting student data, and monitoring student progress. Although not specific to PRT protocol, these concerns provide intervention developers with important information regarding the types of resources teachers need. Development and testing of training materials for teachers and staff, parent information, and data collection materials that fit the context of the classroom and school environment are essential for the sustainability of interventions in the community. Responding to these specific teacher needs is a critical step in effective translation of EBPs. 

### 4.2. Additional Research

Teachers reported particular difficulty with and lack of value for the turn taking component. This suggests a need for additional research on this component as part of the translation process. Next steps may involve evaluating how modifications to the component affect overall efficacy of PRT on child behavior and outcome. First, the specific factors that make this component difficult for teachers to implement should be outlined. For example, the turn taking component of PRT requires that the teacher both model a behavior (typically language or play) and present a contingent opportunity to respond to the student in the same interaction. This may be difficult for teachers because they typically demonstrate new skills for students in an instructional manner at the beginning of an interaction, but do not model new skills by taking turns with students once an activity has begun (e.g., they may do the first problem of a math worksheet on the whiteboard as an example, but then students work independently to complete the remaining problems). Empirically identifying how providing an initial demonstration (rather than modeling in the context of a turn) affects student learning would inform the process of possible modification of turn taking for future classroom use. Additionally, unlike other individual components of PRT, turn taking has not been studied in isolation to determine its influence on child behavior within the larger framework of the intervention. This stands in contrast to other components, such as utilizing contingent and direct reinforcement [46, 47], interspersing maintenance tasks [48, 49], and reinforcing attempts at correct responding [49, 50], which have all been experimentally examined individually. Teachers' reported difficulty with this component and the current lack of explicit empirical support make turn taking an excellent component for experimental examination and potential modification in order to make PRT optimally usable for classroom teachers. 

A second component for which teachers expressed difficulty and lack of value is the multiple cues component. Teachers indicated that the description of the component was difficult to understand and expressed concern that the multiple cues component may not be developmentally appropriate for all of their students with ASD. 

A review of the literature indicates a relationship between developmental level and overselectivity (i.e., responding to simultaneous multiple cues) and reveals that typically developing children do not consistently respond to multiple cues until 36 months of age [51]. Because the age of reliable ASD diagnosis has continued to drop [52], it is likely that students receiving services in today's classrooms are too young to be reliably expected to respond to conditional discriminations. In addition, the majority of studies on overselectivity and ASD were conducted twenty to thirty years ago [53]. It is likely that children receiving services for ASD today are distinct from the populations used in older studies. The lack of current research on overselectivity and ASD leaves open the question of whether conditional discriminations are a difficulty for students today, and thus whether teachers need to specifically teach this skill. An examination of the extent to which today's children with ASD struggle with conditional discriminations may help to shape recommendations for the use (or omission) of this PRT component in the classroom. 

### 4.3. Limitations

There are several limitations to these data that must be acknowledged. First, these data consist of teacher reports of how they use PRT in the classroom. Observational data of teacher use of PRT and fidelity of implementation were not obtained from these teachers. We relied on teacher report of training as it was obtained in this community sample and do not have details regarding the quality of training or level of implementation of PRT by these teachers. It is possible that teachers may differ in their ease of use of PRT depending upon their level of training and competency in the model [54]. However, it is likely that the type of training reported by participating teachers represents typical levels of training in EBPs in public educational settings. In addition, a recent study examining teacher use of PRT in 21 classrooms in two different cities supports the idea that teachers do not consistently use turn taking or multiple cues and are best able to implement antecedent strategies such as providing a clear cue with fidelity [55]. Second, data were obtained from a small group of teachers in Southern California classrooms who represent only a subset of teachers contacted. Most teachers were Caucasian and almost had advanced degrees, which likely does not reflect the demographics of the majority of teachers. Specific benefits and barriers to the use of PRT may be different across the country and should be explored in future research. 

### 4.4. Conclusions

Overall, the results of this investigation indicate that teachers perceive PRT as an intervention that is useful and practical for classroom use. Teachers valued many of the individual components of PRT, as well as the naturalistic behavioral principles that provide its theoretical foundation. Consistent teacher feedback across focus groups indicates areas for possible adaptation and further study. Components the teachers value but report to be difficult are likely to require additional training rather than radical adaptation. This perception of implementation difficulty may reflect teacher's lack of confidence in implementing these strategies. This may not be specific to PRT but may reflect a general dearth of intensive training in autism interventions in general. However, for the components that teachers report both not valuing and not using, additional research may be needed to identify possible modifications and determine the relative importance of these components for the effectiveness of PRT. Closer empirical examination of these components may benefit not only translation of PRT to classrooms but may also be important to PRT as a whole. These findings are useful in determining next steps for researchers interested in systematic adaptation of PRT for the classroom as well as clinicians, such as teachers, who provide frontline services to children with ASD. 

 Overall, this research may serve as a model for the process of adapting EBPs for clinical settings more generally. A phased approach, in which (1) teacher feedback is solicited, (2) specific adaptations are studied empirically in the laboratory, and then (3) appropriate adaptations are tested in the field, may assist with effective translation of EBPs into the community. It is clear that unidirectional models of translating EBPs for clinical use are insufficient and ineffective. Providing the opportunity for an interactive exchange of information between researchers and teachers is a likely first step toward effective translation of EBPs. Additionally, the process of gathering information about what works in educational settings should improve the quality of resources resulting from systematic scientific adaptation. A model for facilitating the widespread delivery of high-quality, EBPs to students with ASD is a necessary and crucial step toward effective treatment of the disorder that warrants the attention of researchers and teachers alike. Using qualitative methods to integrate teachers' opinions and values into the research process may enhance dissemination.

## Figures and Tables

**Table 1:** Summary of PRT components and focus group feedback.

PRT component/area of need	Definition	Focus group feedback
Gains attention	Teacher must have the student's attention before presenting an opportunity.	(i) Important, but easy to forget (ii) Difficult to ensure all students are attending in a group (iii) Can be difficult with ASD students in general

Clear opportunity/instruction	The question/opportunity must be clear and appropriate to the task.	(i) Easy to implement consistently

Maintenance tasks	Tasks that are easy (maintenance) must be interspersed with more difficult tasks (acquisition).	(i) Difficult to identify for each student (ii) Minimizes frustration (iii) Difficult to train paraprofessionals

Child choice (shared control)	The teacher should follow the student's choice of tasks, to a large extent, and/or provide choices within tasks.	(i) Important for maintaining student motivation (ii) Difficult to address some goals with student-chosen materials (iii) Not appropriate in all classroom settings/activities

Turn taking (shared control)	Teacher should model appropriate behavior in the context of a give-and-take interaction with the student.	(i) Difficult to implement, especially in group settings (ii) Difficult in nonplay-based activities (iii) Sometimes not appropriate

Multiple cues	Some instructions should involve cues that include multiple components (two or more aspects of the environment, stimuli, or activity).	(i) Challenging to consistently have multiple cue materials available (ii) Description of multiple cues in the manual is confusing (iii) May not be appropriate for children with minimal language

Contingent consequence	Reinforcement must be contingent on the child's behavior.	(i) Part of general good teaching (ii) Challenging when the behavior is correct but not at an appropriate time

Direct reinforcement	Reinforcement should be natural and directly related to the desired behavior.	(i) Highly effective (ii) Some children only work for edibles or tangible reinforcers (iii) Can be difficult to find for every skill, especially academics

Reinforcement of attempts	Goal-directed attempts to respond must be reinforced.	(i) Useful strategy for keeping motivation high

Training	—	(i) Better training materials/manual needed (ii) Prerequisite knowledge of ABA necessary (iii) Breakdown of components for paraprofessionals needed (iv) Specific techniques for working in groups needed

Resources	—	(i) How to integrate PRT with other strategies (ii) Individualizing for each student (iii) How to use PRT with IEP goals/curriculums (iv) Data collection system (v) Information/handouts for parents

**Table 2:** Summary of recommended adaptations based on teachers' perspectives and quality of classroom implementation.

PRT component/area	Teacher judgments		Possible adaptation
	Importance	Ease of implementation	
Gains attention	High	Med	No adaptation necessary; manual to include strategies for maintaining attention

Clear opportunity/instruction	High	High	No adaptation necessary

Maintenance tasks	High	Low	No adaptation necessary; Training resources and method for identification needed

Child choice (shared control)	High	Med	No adaptation necessary; resources for addressing varied goals with students' chosen items across settings/activities

Turn taking (shared control)	Low	Low	Additional research needed

Multiple cues	Low	Low	Additional research needed

Contingent consequence	High	Med	No adaptation necessary; additional resources and training needed as step is backed by extensive research

Direct reinforcement	High	Low	Additional research needed

Reinforcement of attempts	High	High	No adaptation necessary

Training	High	Low	Training materials needed

Resources	High	N/A	Parent resources needed

Resources	High	N/A	Data collection resources needed

## Appendix

### Sample Focus Group Questions

Vignette Excerpt Alexander is a 4-year-old diagnosed with autistic disorder and mild developmental delay. He is using single words and pointing to communicate his needs. He asks for bubbles and a variety of other items. He is repeating words he hears within 3-4 word sentences and has a speaking vocabulary of at least 40 words; however, he usually uses 1-2 word phrases when he speaks spontaneously. Alexander is able to follow simple commands without cues such as “sit down”. He can point to a variety of pictures and can identify body parts via pointing. Alexander has difficulty relating to people in his environment. He is a very cautious, shy little boy with difficulty separating from his parents. He enjoys simple toys such as busy boxes and puzzles, and a spinning train. He is not yet engaging in symbolic play on his own but will feed a doll when asked to do so. His preferred activities are somewhat stereotypical in nature. Alexander has been observed to engage in some handflapping, especially when excited. Alexander has difficulty with transitions and changes in plans. He is also somewhat distractible but can complete a task when redirected. He is able to tolerate structured sitting with minimal cues for redirection.

(1) What type of program would you set up for this child if he came to your program?
  (a) What specific techniques or type of technique might you use, if any, with this child?
*Presentation of Strategies Used in PRT*
(2) Please review the specific strategies that are used in PRT. Please tell us if you use any of these strategies in your program.
  (a) Would you use any of these strategies with the child in the vignette?
   (b) Would you adapt any of the strategies or techniques for this child? That is, how might your use of the particular technique for this child be different from what the “manual” says?
  (c) In what settings might you use these techniques (if at all)?
(3) Please tell us about techniques you really like to use in your classroom (both those listed as part of PRT and those not listed). Why do you like them? How have you found them helpful in your program?
(4) Please tell us about any of the listed PRT strategies/techniques you DO NOT like. Why do not you like them?
(5) Please tell us about any techniques (PRT based or otherwise) that you have tried and discontinued. What prompted you to discontinue the technique(s)?
(6) What would you like to say to researchers about how to best help school teachers use evidence-based strategies in classroom programs?
